# The Antibiotic Doxycycline Impairs Cardiac Mitochondrial and Contractile Function

**DOI:** 10.3390/ijms22084100

**Published:** 2021-04-15

**Authors:** Rob C. I. Wüst, Bram F. Coolen, Ntsiki M. Held, Mariah R. R. Daal, Vida Alizadeh Tazehkandi, Luciënne Baks-te Bulte, Marit Wiersma, Diederik W. D. Kuster, Bianca J. J. M. Brundel, Michel van Weeghel, Gustav J. Strijkers, Riekelt H. Houtkooper

**Affiliations:** 1 Laboratory Genetic Metabolic Diseases, Amsterdam Gastroenterology, Endocrinology, and Metabolism, Amsterdam Cardiovascular Sciences, Amsterdam UMC, University of Amsterdam, 1105 AZ Amsterdam, The Netherlands; n.m.held@amsterdamumc.nl (N.M.H.); vida.alizadehtazehkandi@student.uva.nl (V.A.T.); m.vanweeghel@amsterdamumc.nl (M.v.W.); 2 Biomedical Engineering and Physics, Amsterdam Cardiovascular Sciences, University of Amsterdam, Amsterdam UMC, 1105 AZ Amsterdam, The Netherlands; b.f.coolen@amsterdamumc.nl (B.F.C.); m.r.daal@amsterdamumc.nl (M.R.R.D.); g.j.strijkers@amsterdamumc.nl (G.J.S.); 3 Laboratory for Myology, Department of Human Movement Sciences, Faculty of Behavioural and Movement Sciences, Amsterdam Movement Sciences, Vrije University Amsterdam, 1081 BT Amsterdam, The Netherlands; 4 Department of Physiology, Amsterdam Cardiovascular Sciences, Amsterdam UMC, VU University Medical Center, 1081 HZ Amsterdam, The Netherlands; l.baks@amsterdamumc.nl (L.B.-t.B.); m.wiersma@amsterdamumc.nl (M.W.); d.kuster@amsterdamumc.nl (D.W.D.K.); b.brundel@amsterdamumc.nl (B.J.J.M.B.); 5 Department of Radiology, Translational and Molecular Imaging Institute, Icahn School of Medicine at Mount Sinai, New York, NY 10029, USA

**Keywords:** doxycycline, mitochondrial function, cardiac contractility, calcium handling

## Abstract

Tetracycline antibiotics act by inhibiting bacterial protein translation. Given the bacterial ancestry of mitochondria, we tested the hypothesis that doxycycline—which belongs to the tetracycline class—reduces mitochondrial function, and results in cardiac contractile dysfunction in cultured H9C2 cardiomyoblasts, adult rat cardiomyocytes, in *Drosophila* and in mice. Ampicillin and carbenicillin were used as control antibiotics since these do not interfere with mitochondrial translation. In line with its specific inhibitory effect on mitochondrial translation, doxycycline caused a mitonuclear protein imbalance in doxycycline-treated H9C2 cells, reduced maximal mitochondrial respiration, particularly with complex I substrates, and mitochondria appeared fragmented. Flux measurements using stable isotope tracers showed a shift away from OXPHOS towards glycolysis after doxycycline exposure. Cardiac contractility measurements in adult cardiomyocytes and *Drosophila melanogaster* hearts showed an increased diastolic calcium concentration, and a higher arrhythmicity index. Systolic and diastolic dysfunction were observed after exposure to doxycycline. Mice treated with doxycycline showed mitochondrial complex I dysfunction, reduced OXPHOS capacity and impaired diastolic function. Doxycycline exacerbated diastolic dysfunction and reduced ejection fraction in a diabetes mouse model vulnerable for metabolic derangements. We therefore conclude that doxycycline impairs mitochondrial function and causes cardiac dysfunction.

## 1. Introduction

In the heart, a high constant energy utilization rate is coupled to constant energy production by mitochondrial oxidative phosphorylation (OXPHOS) [1,2,3,4]. Mitochondria therefore serve a crucial role for optimal cardiac functioning, and they sustain their physiological role by forming physically connected (sub)networks that provide a rapid conductive path for energy distribution [5].

An important feature of mitochondria is their bacterial ancestry [6]. Throughout evolution, the vast majority of genes encoding mitochondrial proteins have moved to the nuclear DNA (nDNA), and these nuclear-encoded proteins need to be imported into the mitochondria. However, mitochondria still retain their own circular DNA (mtDNA), encoding the mitochondrial 16S and 12S rRNA, 22 tRNAs and 13 core subunits of the OXPHOS system, including seven crucial subunits of mitochondrial complex I [7,8]. The stoichiometric balance between both genomes is tightly regulated, and this mitonuclear protein balance is crucial for optimal mitochondrial function [9,10].

Certain classes of antibiotics block protein synthesis in bacteria and thereby limit bacterial growth. However, because of the evolutionary similarities between the bacterial and mitochondrial protein synthesis machinery, these antibiotics may also inhibit normal mitochondrial function [10,11,12] and negatively affect cardiac contractility. Other antibiotic classes, for instance, penicillins, affect bacterial cell wall integrity and do not possess the same potential side effects.

A link between disturbed mitochondrial protein synthesis and cardiac dysfunction is likely, because rare inherited disruptions of mitochondrial translation result in heart failure, dilated and/or hypertrophic cardiomyopathy [13,14,15]. Additionally, cardiac and cardiovascular dysfunction is an important clinical symptom in patients with pathogenic mtDNA mutations [16]. Although tetracyclines are known to pharmacologically inhibit mtDNA translation by binding to the 30S subunit of the mitochondrial ribosomes, it is currently unclear whether tetracycline-induced mitochondrial dysfunction also occurs in the heart, affecting mitochondrial and contractile function in cardiomyocytes and animals.

Large cohort studies have tried to link antibiotic use and adverse cardiovascular outcomes. For instance, clindamycin and doxycycline inhibit mitochondrial protein synthesis and cause mitochondrial dysfunction [17], with an increased risk of cardiac malformations during pregnancy [18]. Similarly, the protein synthesis inhibitors azithromycin and clarithromycin are associated with an increased risk of arrhythmias, myocardial infarctions and death in patients with cardiovascular disease [19,20]. The underlying mechanism remains unclear, as mitochondrial metabolism was not studied before.

Here, we used doxycycline as a prototypical tetracycline antibiotic [12]. Doxycycline is commonly prescribed for long-term use in patients with acne, sexual transmitted diseases, urinary tract infections and abdominal aortic aneurysms [21]. Tetracyclines are used extensively in tetracycline-controlled transcriptional activation studies [22]. Blood doxycycline concentrations between 5 and 15 μg/mL have been reported in humans [23,24], but can accumulate in tissue several-fold [25]. Detailed pharmacokinetics after long-term use in humans are unknown.

We hypothesized that tetracycline toxicity has negative consequences for cardiac mitochondrial and contractile function in intact and permeabilized H9C2 cardiomyoblasts, investigated the changes in metabolic flux distribution, and visualized mitochondrial networks. We measured cytosolic calcium transients in electrically stimulated adult rat cardiomyocytes and studied the arrhythmogenic potential of doxycycline in *Drosophila melangaster*. Ultimately, magnetic resonance (MR) imaging was used to assess cardiac contractile function in mice and in an animal model of metabolic derailment (db/db) treated with doxycycline, as we expected that particularly metabolically vulnerable animals are more likely to suffer from contractile dysfunction upon doxycycline treatment.

## 2. Results

### 2.1. Mitonuclear Protein Imbalance in Doxycycline-Treated Cardiac Cells

**Figure 1:**
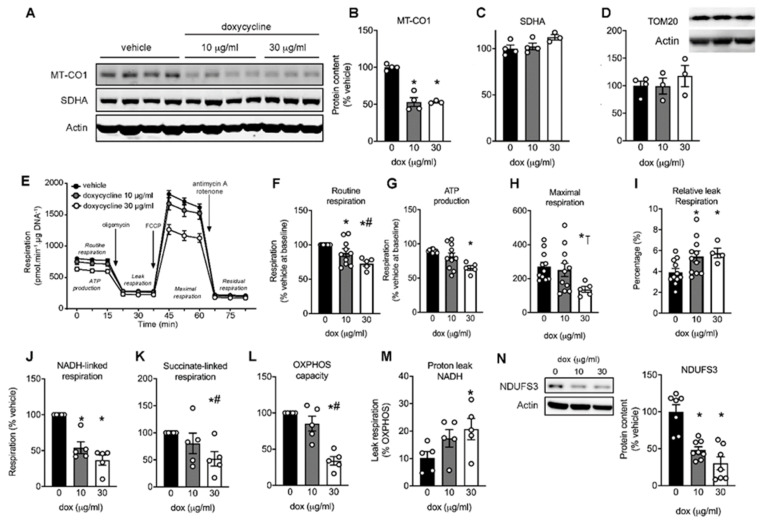
Mito-nuclear protein imbalance and mitochondrial complex I dysfunction in H9C2 cardiomyoblasts exposed to doxycycline. H9C2 cardiomyoblasts were exposed to doxycycline at 0, 10 or 30 μg/mL for 72–96 h. (**A**) Western blot showing protein expression of mitochondrially-encoded cytochrome c oxidase I (MT-CO1) and nuclear-encoded succinate dehydrogenase complex, subunit A (SDHA). Actin was used as a loading control. (**B**) MT-CO1 protein content (normalized to vehicle) was significantly lower in doxycycline-treated cells. (**C**,**D**) SDHA and TOM20 protein content is unaffected by doxycycline. (**E**) Typical Seahorse run with injections of oligomycin, carbonylcyanide-4-(trifluoromethoxy)-phenylhydrazone (FCCP), antimycin A and rotenone highlighted. (**F**–**H**) Routine, oxygen consumption used for ATP production (Routine-Leak respiration) and maximal respiration was reduced in doxycycline-treated cells. (**I**) Normalized leak respiration (i.e., Leak / Maximal respiration) was higher in both concentrations of doxycycline. (**J**) Mitochondrial complex I-stimulated respiration (with substrates pyruvate and malate) was significantly reduced in cells exposed to 10 or 30 µg/mL doxycycline compared to vehicle. (**K**) Complex II-stimulated (with succinate and rotenone) was only reduced at 30 µg/mL doxycycline compared to vehicle or low dose doxycycline. (**L**) Oxidative phosphorylation (OXPHOS) capacity (with pyruvate, malate and succinate) dose-dependently decreased following doxycycline exposure, note that panel H shows maximal uncoupled respiration. (**M**) Relative leak respiration (after oligomycin) with complex I substrates showed increased proton leak from complex I at 30 µg/mL doxycycline. No differences were observed with other substrates. (**N**) Western blot analysis revealed a large reduction in protein content of nuclear-encoded mitochondrial complex I subunit NDUFS3 in doxycycline-treated H9C2 cells. Bar graphs are expressed as mean±SEM. *: *p* < 0.05 vs. vehicle, one-way ANOVA followed by Tukey’s post-hoc test for multiple comparisons, #: *p* < 0.05 vs. 10 µg/mL doxycycline. ⊺: *p* = 0.088 vs. 10 µg/mL doxycycline.

To establish the effect of tetracycline antibiotics on cardiomyocyte mitochondrial function, we used the H9C2 rat cardiomyocyte cell line, and used doxycycline as a prototypical antibiotic of the tetracycline class. We first tested whether low (10 µg/mL) and high (30 µg/mL) doses of doxycycline induced mitonuclear protein imbalance [10], i.e., a lower protein abundance of mtDNA-encoded cytochrome c oxidase subunit I (MTCO1) given its inhibitory effect on mitochondrial translation. Indeed, MTCO1 protein content was significantly lower in cells treated with doxycycline for up to 96 h compared to vehicle-treated controls (Figure 1A,B). The protein abundance of the nuclear-encoded succinate dehydrogenase complex subunit A (SDHA) was unchanged (Figure 1A,C). To further investigate whether doxycycline affects the overall protein content of mitochondrial proteins—also as a reflection of mitochondrial abundance—we measured the protein content of a widely used mitochondrial marker, translocase of outer-membrane 20 (TOM20). Similar to SDHA, the expression of TOM20 was unaffected (Figure 1D). Together, the significantly lower MT-CO1 expression, coupled to unchanged SDHA and TOM20 is a clear indication of a mitonuclear protein imbalance in doxycycline-treated H9C2 cardiomyoblasts.

### 2.2. Maximal Mitochondrial Respiration Is Lower in Doxycycline-Treated Cardiac Cells

We performed respirometry to test whether doxycycline reduced mitochondrial respiration (Figure 1E). Doxycycline exposure resulted in a dose-dependent reduction in routine mitochondrial respiration (Figure 1F), ATP-linked respiration (Figure 1G) and maximal uncoupled respiration (Figure 1H). The relative leak respiration (i.e., leak/maximal respiration), which is an indication for OXPHOS uncoupling, was higher in doxycycline- versus vehicle-treated cells (Figure 1I). These data suggest that mitochondrial respiration was negatively affected in H9C2 cardiomyoblasts treated with doxycycline.

### 2.3. Doxycycline Reduced Both Complex I- and Complex II-Driven Respiration in Cardiac Cells

The equal expression of SDHA and TOM20 (Figure 1A,D) indicated that the lower respiration might be attributed to qualitative mitochondrial defects rather than a lower number of mitochondria *per se*. Mitochondrial DNA encodes seven complex I subunits but no complex II subunits, and as such, complex I respiration might be more affected by doxycycline treatment compared to complex II respiration. To test this and to further pinpoint the underlying cause of the lower mitochondrial respiration in doxycycline-treated H9C2 cardiomyoblasts, we measured mitochondrial respiration in digitonin-permeabilized cells. Mitochondrial respiration with mitochondrial complex I substrates malate and pyruvate was significantly lower at both concentrations of doxycycline (Figure 1J). Mitochondrial respiration with the complex II substrate combination succinate/rotenone was only significantly reduced at the highest doxycycline concentration (Figure 1K). Similar effects were obtained for OXPHOS capacity with both complex I and II substrates present (Figure 1L). When normalizing leak respiration with complex I substrates for maximal respiration, we observed a dose-response relationship in the relative proton leak, indicative of more uncoupling, in doxycycline-treated cardiomyoblasts compared to control (Figure 1M). Relative proton leak for complex II and I+II substrates were not different between conditions (data not shown). These data indicate that complex I respiration was more affected compared to complex II respiration, in agreement with the contribution of mitochondrial DNA for producing critical complex I subunits. The involvement of mitochondrial complex I in the mitochondrial toxicity of doxycycline was further shown by a reduction in protein content of the nuclear-encoded complex I subunit NDUFS3 in doxycycline-treated cells (Figure 1N).

### 2.4. Doxycycline-Treated Cardiomyocytes Shift Away from Oxphos towards Glycolysis

**Figure 2:**
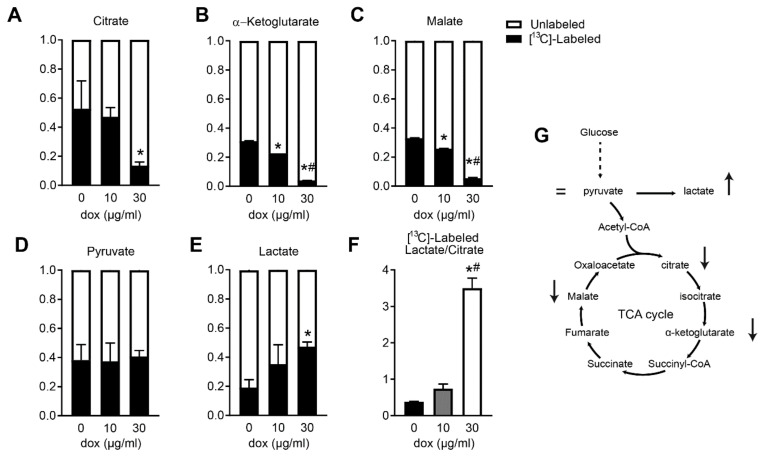
Reduced glucose flux into the tricarboxylic acid (TCA) cycle in H9C2 cardiomyoblasts exposed to doxycycline. (**A**–**C**) Doxycycline resulted in a lower percentage of incorporation of [^13^C_6_]-labeled glucose into tricarboxylic acid (TCA) cycle intermediates citrate, α-ketoglutarate, and malate. (**D**) Incorporation into pyruvate (both part of the glycolysis and TCA cycle) did not change. (**E**) More incorporation of [^13^C_6_]-labeled glucose in intracellular lactate after doxycycline. (**F**) Ratio of [^13^C]-labeled lactate/citrate increased after doxycycline treatment, indicating a metabolic shift from TCA cycle to glycolysis. (**G**) A graphical overview of the positive or negative changes in glycolytic and TCA cycle intermediates after doxycycline exposure. Bar graphs are expressed as mean±SEM. *: *p* < 0.05 vs. vehicle; #: *p* < 0.05 vs. 10 µg/mL doxycycline, one-way ANOVA followed by Tukey’s post-hoc test for multiple comparisons.

Since we observed a lower oxygen consumption used for ATP production, we performed ^13^C fluxomics using stable isotope-labeled [^13^C_6_]-glucose, and measured the concentrations of intermediary metabolites to further establish the consequent metabolic adaptations. We observed that H9C2 cardiomyoblasts exposed to doxycycline displayed reduced incorporation of labeled glucose into various tricarboxylic acid (TCA) cycle intermediates (Figure 2A–C). Incorporation into pyruvate, which forms the branch point between glycolysis and the TCA cycle, did not change (Figure 2D). As pyruvate can be converted to lactate in the cytosol through anaerobic glycolysis, synthesis of intracellular labeled [^13^C_3_]-lactate levels dose-dependently increased upon doxycycline exposure (Figure 2E). The metabolic switch from mitochondrial oxidative metabolism towards glycolysis (ratio labeled lactate and labeled citrate) was markedly increased especially after high-dose doxycycline (Figure 2F,G). These data indicate that doxycycline resulted in less glucose contribution into TCA cycle intermediates, but more production of lactate, the end-product of glycolysis.

### 2.5. Mitochondria Appear Fragmented In Doxycycline-Treated Cardiac Cells

**Figure 3:**
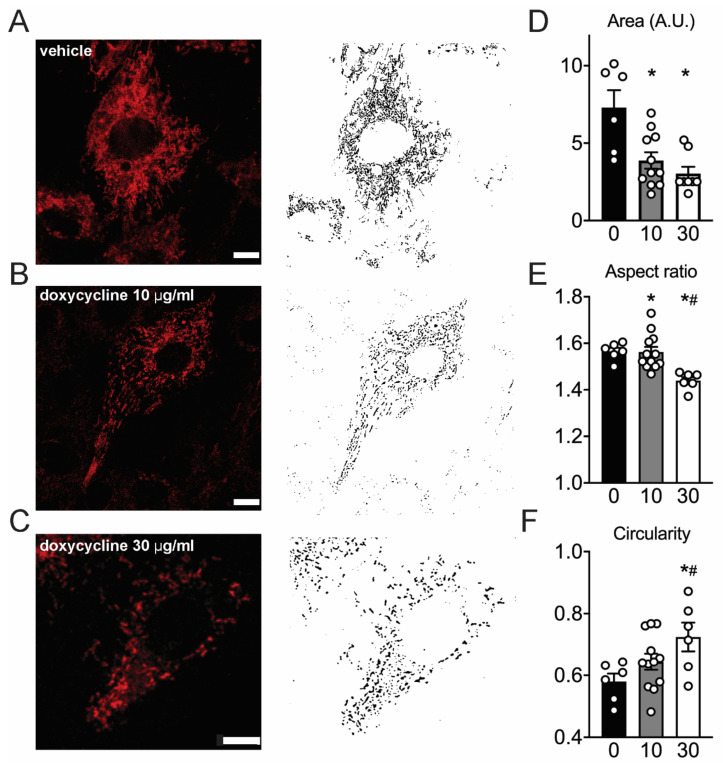
Mitochondria appear more fragmented in doxycycline-treated H9C2 cells. (**A**–**C**), representative H9C2 cells loaded with TMRM with corresponding masks for quantification. (**D**–**F**), Mitochondria in doxycycline-treated H9C2 cells appeared more fragmented, were smaller, had lower aspect ratios and higher circularity compared to vehicle. Values are median (area and aspect ratio) or 25% percentile values (circularity) because of non-normal distribution. *n* = 5–12 independent samples per group on four different days. Error bars are SEM. Scale bar: 10 μm. *: *p* < 0.05 vs. vehicle; #: *p* < 0.05 vs. 10 µg/mL doxycycline, one-way ANOVA followed by Tukey’s post-hoc test for multiple comparisons.

In a healthy situation, mitochondria form a reticulum that allows OXPHOS to function optimally. Conversely, mitochondrial stress often leads to mitochondrial fragmentation [12,26]. Therefore, we tested whether mitochondria retained their network structure after doxycycline treatment. Mitochondria in live H9C2 cardiomyoblasts were imaged with the dye TMRM, and appeared more fragmented in doxycycline-treated cells (Figure 3A–C). Quantification of these ultrastructural changes indicates that mitochondrial area in untreated cells was significantly larger compared to doxycycline (Figure 3D). In accordance, the aspect ratio was lower and the circularity was higher in doxycycline-treated cells (Figure 3E–F). These data are consistent with the observation of a fragmented mitochondrial network in doxycycline-treated H9C2 cardiomyoblasts.

### 2.6. Doxycycline-Treated Adult Rat Cardiomyocytes Have Higher Diastolic Calcium and More Irregular Calcium Waves

**Figure 4:**
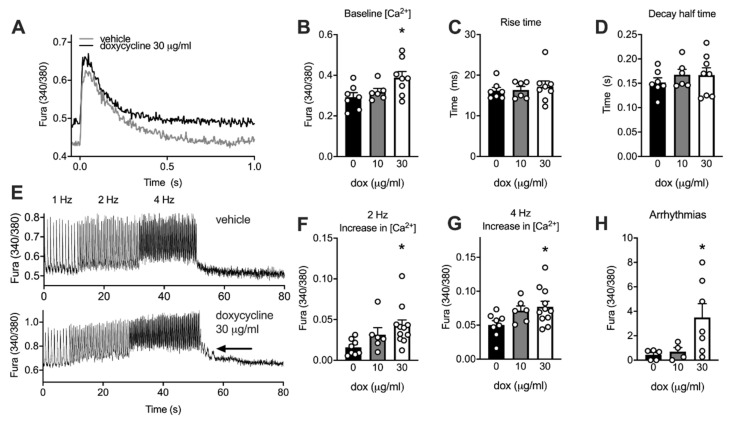
Higher resting cytosolic calcium and more arrhythmias in adult cardiomyocytes exposed to doxycycline. (**A**) Typical examples of calcium transients (340/380 ratio) obtained from adult rat cardiomyocytes exposed maximal 48 h to doxycycline, electrically stimulated at 1 Hz. (**B**) Baseline cytosolic Ca^2+^ concentration was significantly higher after exposure to 30 µg/mL doxycycline compared to vehicle. (**C**–**D**) No differences were observed in the shape of the calcium transient. (**E**) Typical examples of calcium transients of vehicle-treated (top) and doxycycline-treated (30 µg/mL; bottom) adult rat cardiomyocytes electrically stimulated at 2 and 4 Hz for 20 s (after a period at 1 Hz). Arrow point to irregular Ca^2+^ waves, quantified in H. (**F**–**G**) At higher stimulation frequencies (2 and 4 Hz), baseline (diastolic) calcium increased to a larger extent in doxycycline-treated cells. (**H**) More arrhythmias were observed in adult cardiomyocytes treated with 30 µg/mL doxycycline during and straight after about 2 and 4 Hz. Bar graphs are expressed as mean±SEM. *: *P* < 0.05 vs. vehicle, one-way ANOVA followed by Tukey’s post-hoc test for multiple comparisons.

We used adult rat cardiomyocytes to establish whether doxycycline-induced mitochondrial alterations affect cardiac contractile function. Cardiomyocytes were kept in culture for maximally 48 h in the presence and absence of doxycycline. Cytosolic calcium transients were measured at 1 Hz to assess contractile Ca^2+^ handling (Figure 4A). Baseline cytosolic Ca^2+^ concentration was significantly higher in the high dose of doxycycline-treated cardiomyocytes compared to control (Figure 4B). The calcium amplitude and maximal cytosolic Ca^2+^ concentration were not different between conditions. No differences were observed in the shape of the calcium transient, as time to peak and decay half time were not significantly different between conditions (Figure 4C,D).

Next, we tested whether cardiomyocytes were able to cope with higher stimulation frequencies as a model of metabolic and contractile stress [27] (Figure 4E–H). Baseline cytosolic Ca^2+^ concentration increased to a larger extent in doxycycline-treated cardiomyocytes at 2 and 4 Hz (Figure 4F,G). Maximal cytosolic Ca^2+^ concentration at 4 Hz was not different between conditions and no other doxycycline-induced changes in the shape of the calcium transient were observed (data not shown). More arrhythmias, i.e., calcium waves and irregular contractions, were counted at high doxycycline concentrations when compared to a low dose or no doxycycline during and up to 30 s after this bout at 2 and 4 Hz (Figure 4H). These data suggest that doxycycline increases the baseline cytosolic calcium concentration and makes the cardiomyocyte more vulnerable to arrhythmias.

### 2.7. Doxycycline Exposure Causes Contractile Dysfunction in Drosophila Melanogaster Hearts

**Figure 5:**
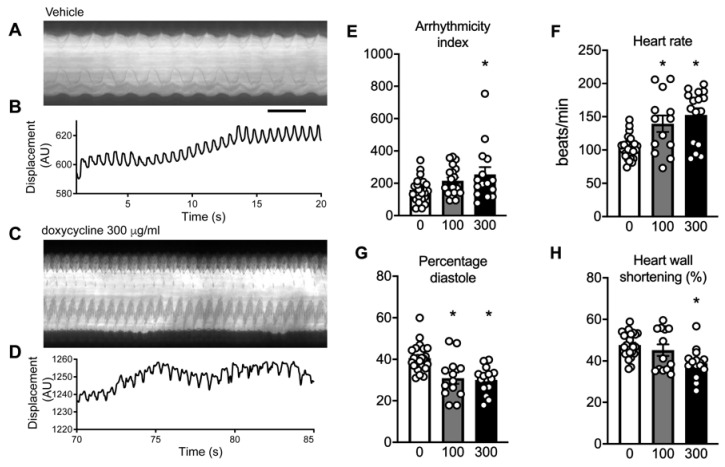
Arrhythmias and contractile dysfunction in the heart of *Drosophila melanogaster* exposed to doxycycline. (**A**–**D**) Typical examples of kymographs and cardiac wall displacements obtained from videos of the cardiac wall in prepupae of *Drosophila melanogaster*. (**E**) Exposure to high-dose doxycycline (300 μg/mL) resulted in a significantly higher arrhythmicity index, indicative of a higher occurrence of arrhythmias compared to vehicle or low concentration of doxycycline (100 μg/mL). (**F**) Basal heart rate was significantly higher at 300 µg/mL doxycycline, with intermediate values at 100 µg/mL. (**G**) The relative time of diastole was shortened, indicative of diastolic dysfunction, after both concentrations of doxycycline. (**H**) Contractile dysfunction was observed as the heart wall shortening was reduced after doxycycline treatment. Bar graphs are mean±SEM. *: *P* < 0.05, one-way ANOVA followed by Tukey’s post-hoc test for multiple comparisons.

To extend these findings to an in vivo model for cardiac function, *Drosophila melanogaster* were exposed to low and high doses of doxycycline (100 and 300 μg/mL respectively; similar as used before [12]), and cardiac heart wall contractions were visualized in prepupae (Figure 5A–D). We observed a markedly higher arrhythmicity index after exposure to high doxycycline concentrations, with intermediate values for the low doxycycline concentration (Figure 5E). This increased arrhythmicity index confirmed the arrhythmogenic substrate of doxycycline. Moreover, doxycycline dose-dependently increased basal heart rate (Figure 5F), and concomitantly, the time for diastole and systole were significantly shorter after doxycycline (data not shown). Moreover, the relative time of diastole in the heart period was shortened after doxycycline exposure (Figure 5G). These results indicate a reduced filling capacity of the heart tube. Finally, the displacement of the heart wall between systole and diastole significantly reduced at high doxycycline concentration (Figure 5H), indicating that doxycycline also impaired systolic function.

### 2.8. Doxycycline Exposure Reduces Mitochondrial Respiration and Results in Contractile Dysfunction in Mice, Particularly in Diabetic Animals

**Figure 6:**
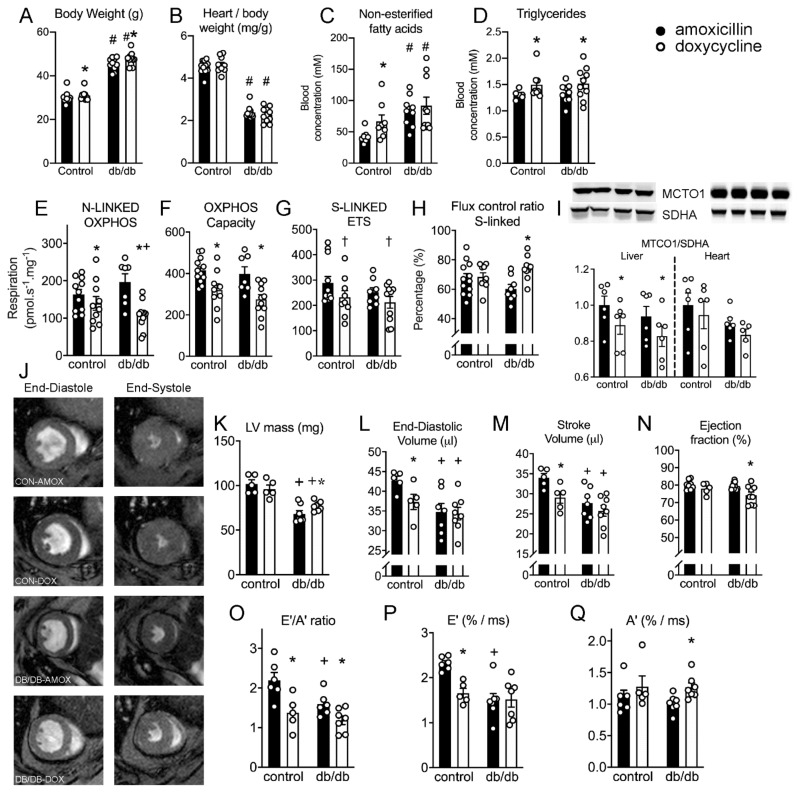
Doxycycline impairs cardiac mitochondrial bioenergetic function, and results in diastolic dysfunction, which was exacerbated in diabetic animals. (**A**,**B**) body and heart weights of control (db+) and diabetic (db/db) mice. (**C**) Fasted non-esterified fatty-acid concentrations were higher in db/db compared to control animals and were significantly higher after doxycycline in control. (**D**) Triglyceride concentration was significant higher after doxycycline exposure in both animal models. (**E**) NADH (complex I)-linked oxidative phosphorylation (OXPHOS) was significantly lower in normal animals, but was reduced to a larger extent in db/db animals. (**F**) Maximal OXPHOS with complex I (NADH-linked) and complex II (succinate) substrates was significantly lower in doxycycline-treated animals compared to amoxicillin-treated animals. (**G**) Maximal succinate-driven complex II respiration with rotenone tended to be lower after doxycycline treatment (*P* = 0.06). (**H**) Normalized respiration (flux control ratio; FCR) for succinate-linked respiration increased in db/db animals treated with doxycycline, but not in healthy animals, FCR for NADH-linked respiration tended to be lower in db/db animals, but not in healthy animals (*P* = 0.15, not shown). (**I**) The protein ratio of MTCO1/SDH was lower in the liver, but not in the heart, after doxycycline exposure in both animal models. (**J**) Representative examples of short axis MRI scans at mid-heart during the end-diastole (left) and end-systole (right). (**K**) Left ventricular (LV) mass was lower in db/db animals and doxycycline significantly increased LV mass only in db/db animals. (**L**,**M**), End-diastolic and stroke volume were lower in control animals exposed to doxycycline, and in db/db animals. (**N**) Ejection fraction was only marginally, but significantly, lower in db/db animals exposed to doxycycline. (**O**–**Q**), Diastolic function (E’/A’) was lower in control animals exposed to doxycycline, due to lower E’ (stiffening of heart wall), and unaltered A’ (atrial function). Diabetic animals already had lower E’/A’ due to lower E’, and doxycycline further lowered E’/A’ due to higher A’. Bar graphs are expressed as mean±SEM. *: *P* < 0.05 vs. amoxicillin; †: *P* = 0.06 vs. amoxicillin; +: *P <* 0.05 vs. corresponding control animal, two-way ANOVA followed by Tukey’s post-hoc test for multiple comparisons.

Mice were treated with doxycycline for three weeks, and amoxicillin was used as a control antibiotic to account for microbiome alterations. We included a group of animals with a metabolic derailment (db/db animals) to test whether these animals are at higher risk to develop cardiac dysfunction after doxycycline exposure. Diabetic mice had higher body weight but lower heart/body weight (Figure 6A,B), had higher fasted glucose (13 ± 3 vs. 43 ± 14 mM in control and diabetic animals) and non-esterified fatty acid blood concentrations (Figure 6C), reminiscent of diabetic cardiomyopathy. Three diabetic mice were found dead prematurely during the doxycycline exposure, but no apparent non-cardiac cause of death could be established.

Body weight was significantly higher in doxycycline-exposed animals (Figure 6A), but heart/body weight was not different between amoxicillin and doxycycline-exposed animals (Figure 6B). Fasted blood glucose levels were not different after doxycycline exposure (12 ± 3 vs. 45 ± 13 mM in control and diabetic animals), but non-esterified fatty acid concentrations were significantly higher, only in doxycycline-exposed control animals (Figure 6C). Triglyceride concentrations were consistently higher after doxycycline in both groups (Figure 6D).

Mitochondrial leak respiration was not different between groups. NADH-linked, complex I-driven, respiration was significantly lower after doxycycline exposure in control mice, and doxycycline exposure caused a greater reduction in diabetic animals (Figure 6E). Maximal oxidative phosphorylation capacity was significantly lower after doxycycline in both animal models (Figure 6F), while succinate-linked respiration, through complex II, only tended to be lower after doxycycline exposure (Figure 6G). Together with the higher normalized succinate-linked respiration (Figure 6H), these results are indicative of a modest mitochondrial complex I dysfunction induced by doxycycline exposure.

We next determined the ratio between protein concentration of MTCO1 and SDHA in the liver and cardiac samples, and observed that the MTCO1/SDHA ratio in only the liver was significantly lower (Figure 6I), likely due to a higher overall protein turnover rate in liver compared to heart.

Next, we determined cardiac function by MR imaging to evaluate whether doxycycline resulted in altered systolic and diastolic function. Typical examples of short-axis scans and volume changes during the cardiac cycle are shown in Figure 6J. Heart rate was not different between groups, and was on average 516 ± 60 beats/min during the cardiac scans. Left-ventricular mass was lower in db/db animals (Figure 6K), in concordance with a lower heart weight. Doxycycline exposure increased left-ventricular mass only in db/db animals (Figure 6K). End-diastolic and stroke volume (Figure 6L,M) were lower in doxycycline-treated control animals compared to amoxicillin, and in both db/db groups compared to control animals. Ejection fraction was not different between control and db/db animals, but was marginally, but significantly, lower in db/db animals exposed to doxycycline (Figure 6N).

Diastolic function was impaired in control animals exposed to doxycycline, evidenced by a lower ratio of peak velocity of the blood flow during the elastic (E’) phase and atrial kick (A’; Figure 6O), due to a lower E’ (Figure 6P) but unaltered A’ (Figure 6Q). This indicates that the relaxation of the heart is impaired due to stiffening of the heart wall during early diastole, rather than impaired atrial function.

Diabetic animals already had lower E’/A’ due to lower E’, confirming diabetic cardiomyopathy with impaired cardiac filling. Doxycycline exposure in diabetic mice reduced E’/A’ further, but in contrast to control animals not due to lower E’, but instead a higher A’ (Figure 6O–Q). These results are indicative of an atrial compensatory mechanism to ensure adequate cardiac filling and stroke volume, despite small reductions in ejection fraction (Figure 6N). Together, the mouse experiments demonstrate that doxycycline exposure impairs mitochondrial bioenergetic function and causes diastolic dysfunction, which exacerbated towards a lower ejection fraction and higher left-ventricular mass in db/db mice.

## 3. Discussion

Tetracycline antibiotics are well-known and widely used antibiotics, and their use is seldom questioned in long-term clinical use or during Tet-on/Tet-off applications. Although its inhibiting effect on mitochondrial protein synthesis has long been acknowledged [11], and even helped scientists unravel the bacterial ancestry of mitochondria [6], very few studies have focused on the possible toxic effects of antibiotics on cell metabolism and function. Cardiomyocytes have a high energetic requirement, and might be prone to doxycycline-mediated toxicity. Here, we report the negative effects of doxycycline antibiotics on mitochondrial function by showing that it particularly reduced OXPHOS and impairs contractile function in various animal models. Indeed, our study provides evidence for tetracycline-mediated alterations in cardiac mitochondrial and contractile function in cultured cells, adult cardiomyocytes, fruit flies and mice.

Upon doxycycline treatment, cardiac mitochondria appeared more fragmented, and maximal mitochondrial OXPHOS capacity was reduced. It should be noted however that TMRM particularly accumulates inside mitochondria with functional inner-membrane potential. While we did not determine the total TMRM signal per cell, the TOM20 and SDHA protein concentrations suggest no major differences in mitochondrial volume density.

In the undifferentiated H9C2 myoblasts, we observed that the ^13^C-labeled glucose was incorporated into the TCA cycle. The lower routine respiration and higher incorporation of ^13^C-labeled glucose into lactate compared to citrate confirmed a glycolytic shift away from OXPHOS in cardiomyoblasts exposed to doxycycline. The lower OXPHOS capacity was partly due to lower respiration and increased proton leak using mitochondrial complex I substrates. Interestingly, not only was the mitochondrially-encoded MT-CO1 lower, also the nuclear-encoded mitochondrial complex I subunit NDUFS3 was significantly lower in doxycycline-treated cells (Figure 1N), but not in the heart of doxycycline-treated mice (data not shown). We anticipate that the assembly pathway for mitochondrial OXPHOS (super)complexes are disrupted, due to the absence of the core subunits encoded by the mitochondrial DNA. The mtDNA encodes 7 out of 46 subunits of complex I [8], and as such, an incomplete assembly of mitochondrial complex I and increased degradation of some complex I subunits after antibiotic treatment has also been shown in HEK293 cells [28]. Genetic mutations in mtDNA-encoded proteins are linked with a disrupted complex I assembly and stability, ultimately leading to respiratory abnormalities [29]. In accordance, we observed that doxycycline reduced complex I respiration to a larger extent compared to complex II respiration in cells and animals. The fact that OXPHOS capacity and complex II respiration are also negatively affected by doxycycline in cells and animals is not surprising, since the mtDNA also encodes for 1–3 subunits in complexes III and IV, which also participate in mitochondrial oxygen consumption. The turn-over rate in mitochondrial proteins is much higher in differentiating cells compared to adult mouse cardiomyocytes in vivo. This likely explains the smaller effects of doxycycline on mitochondrial and nuclear-encoded proteins in doxycycline-treated mice, despite functional impairments in mitochondrial respiration. Future studies are required to study differences in supercomplex formation in doxycycline-exposed cultured cells and animals.

OXPHOS impairments have been extensively linked to contractile dysfunction in the heart, particularly during elevated heart rates [2]. It is a long-held paradigm that the energetic deficit plays a causal role in the progression of chronic heart failure [30,31]. Patients with a genetic mtDNA mutation (such as MELAS patients [32]), but also those with fatty acid oxidation defects [33] suffer from cardiac dysfunction. The importance of mitochondrial complex I for the support of fatty acid oxidation further establishes this link, as impaired complex I respiration can also inhibit fatty acid oxidation [33,34]. As such, cardiac energetics is a crucial component of optimal excitation-contraction coupling [2,3,31].

We observed higher diastolic calcium levels in adult cardiomyocytes exposed to doxycycline and cardiomyocytes and *Drosophila* were more susceptible to arrhythmias. We subsequently observed mitochondrial and diastolic dysfunction in mice treated with doxycycline. These dysfunctions were exacerbated in animals with pre-existing type 2 diabetes mellitus, indicative that doxycycline might be a second-hit for mitochondrial and cardiac dysfunction in animal models with pre-existing metabolic vulnerability.

A higher resting cytosolic calcium has been linked to an increased cross-bridge recruitment and concomitant diastolic stiffness [35]. We hypothesize that an impaired ATP-dependent Sarco/Endoplasmic Reticulum Ca^2+^-ATPase (SERCA) activity [36,37], underlies this increased cytosolic calcium concentration. The impaired calcium handling and mitochondrial bioenergetic function likely increase local ADP concentration, which affects cross-bridge recruitment [35], slowed cardiac filling time and diastolic dysfunction in intact hearts [35,38] in *Drosophila* and mice. An increased intracellular calcium concentration can also contribute to a fragmented mitochondrial network observed in our cultured cells [39].

Mitochondrial dysfunction results in increased susceptibility to cardiac arrhythmias [40,41,42]. We found a higher incidence of calcium waves and irregular contractions (indicative of arrhythmias) in adult cardiomyocytes and flies after doxycycline exposure. Various metabolic pathways underlie this susceptibility to cardiac arrhythmias. Intracellular ion homeostasis can be impaired through ADP/ATP-induced opening of the sarcolemmal K_ATP_ channels [43], and/or ryanodine receptors become leaky through reactive oxygen species or cellular redox alterations [44], both affecting membrane excitability and mitochondrial and cytosolic calcium handling. While we did not observe altered calcium dynamics in our isolated cardiomyocytes, we did not assess sarcoplasmic reticulum calcium load, sodium or potassium fluxes. As such, the causal molecular mechanism underlying this link between doxycycline and cardiac arrhythmias is currently unknown.

Elucidating the long-term effects of antibiotics and their downstream mechanistic consequences on cardiac metabolism ultimately affect clinical heart failure management. The antibiotic-induced inhibition of mitochondrial protein synthesis could provide mechanistic insights into understanding the putative links between certain antibiotics and cardiac events [17,18,19,20]. Interestingly, previous studies have observed that doxycycline is beneficial in the management of patients after ST-Elevation Myocardial Infarction (STEMI) [45]. Likely this effect is due to the known inhibition of matrix metalloproteinases (MMPs) by doxycycline [46]. Interestingly, the diastolic dysfunction we observed in the *Drosophila* and mice could not be explained by a doxycycline-induced inhibition of MMPs. Here, we describe that doxycycline altered mitochondrial metabolism, contributing to cardiac metabolic dysfunction. However, exposure to doxycycline has more intracellular effects, independent of the altered cardiac metabolism described here. As doxycycline is a calcium and iron chelator, it even has been shown to inhibit mitochondrial swelling [47]. Of note here as well is that doxycycline likely increases food intake and affects systemic inflammation [48], which could be the underlying cause of the very modest, but significantly higher body weight in the mice. It is unknown if the sucrose added to the drinking water to hide the taste of doxycycline contributed to the higher body weight.

One limitation of our study is that we cannot exclude that db/db mice have altered doxycycline metabolism compared to their control counterparts. Circulating doxycycline concentrations greatly depend on whole body uptake and clearance pharmacokinetics, which are difficult to monitor due to fluctuating blood concentrations throughout the day [24,25]. Additionally, pharmacokinetics greatly differ between and within animal models due to differences in whole-body metabolism and renal blood flow, to name a few factors. As such, cardiac intracellular doxycycline concentrations can be different between the various cell and animal models tested here. Clinical doses of doxycycline typically result in blood serum concentrations of 5–15 mg/L in humans, but can accumulate in tissue several-fold [23,24,25]. We did not test the doxycycline concentration in the tissues in our study, but the concentration used here likely exceeds the concentrations upon clinical exposure. More research on prolonged doxycycline exposure, and its pharmacokinetics is warranted. Our primary aim was however not to provide evidence of cardiac alterations using clinically relevant doxycycline concentrations, but to provide proof-of-concept that doxycycline exposure can cause metabolic and functional alterations in the heart. Nonetheless, because of the negative effects of doxycycline on cardiac metabolism, we anticipate that in particular patients with an impaired mitochondrial function, such as those with insulin resistance [49,50] or inborn errors of metabolism [51], might be at greater risk of cardiac abnormalities after long-term treatment with certain classes of antibiotics. Additionally, patients at risk of arrhythmias and those with heart failure might need to be careful with long-term use of tetracycline antibiotics. Large-scale clinical studies should be performed to study this in more detail.

In conclusion, treatment with the tetracycline antibiotic doxycycline is detrimental for mitochondrial and contractile function in cultured and ex vivo adult cardiomyocytes, in *Drosophila* and in mice. Due to its cardiotoxicity, the long-term use of tetracyclines should be treated with caution. Future work should identify possible cellular mechanisms that could alleviate these effects.

## 4. Materials and Methods

### 4.1. Cells

Undifferentiated H9C2 cardiomyoblasts were cultured in Dulbecco’s modified Eagle’s medium (DMEM; Lonza, Verviers, Belgium) containing 10% (*v*/*v*) fetal calf serum (Bodinco, Alkmaar, The Netherlands), 25 mM HEPES, 100 μg/mL ampicillin and 100 μg/mL carbenicillin in a humidified atmosphere of 5% CO_2_ at 37 °C. Ampicillin and carbenicillin were used instead of the standard penicillin-streptomycin, since streptomycin is also a bacterial protein synthesis inhibitor, and could have similar mitochondrial side effects. Doxycycline was added for 72–96 h at 10 or 30 μg/mL. Results were compared to vehicle-treated cells.

### 4.2. Western Immunoblotting

Proteins were extracted in protein extraction buffer containing 25 mM Tris-HCl, 150 mM NaCl, 1% (*v*/*v*) NP-40, 1% sodium deoxycholate and 0.1% SDS. Ten-fifteen μg of total protein lysate was loaded on pre-cast 4–15% or 8–16% gradient Criterion TGX SDS-PAGE gels and proteins were transferred onto nitrocellulose membranes and blocked for one hour with 5% nonfat dried milk powder. Antibodies were purchased from Abcam (Cambridge, UK) and diluted 1:1000, unless otherwise stated. Antibodies against MT-CO1 (ab14705), SDHA (ab14715), Tom20 (pab77631, Covalab, Bio-connect, Huissen, Netherlands) were used for immunoblotting. Anti β-tubulin or anti-actin (clone AC-40, Sigma, 1:5000) were used to correct for unequal loading. No loading control was needed for the MTCO1/SDHA ratio. Membranes were washed with TBS-Tween and incubated with horseradish peroxidase-conjugated secondary antibody (goat anti-mouse or goat anti-rabbit; DAKO, Heverlee, Belgium; 1:5000).

### 4.3. Mitochondrial Respiration

Measurements of cellular oxygen consumption were performed using an XFe96 Extracellular Flux Analyzer, using the Seahorse XF Mito Stress Test kit (Seahorse Bioscience, North Billerica, MA, USA). H9C2 cardiomyoblasts were incubated for 5–6 days in culture medium in the presence or absence of doxycycline, followed by overnight incubation at 25,000 cells/well in Seahorse 96-well culture microplates. One hour before measurement, medium was replaced by DMEM (Sigma, D5030, Zwijndrecht, Netherlands) containing 25 mM glucose (Sigma), 1 mM sodium pyruvate (Lonza, Basel, Switzerland), and 2 mM L-glutamine (Life Technologies, Bleiswijk, The Netherlands) and cells were incubated in a non-CO_2_ 37 °C incubator. Mitochondrial respiration was measured before (routine respiration) and after 1.5 μM oligomycin (leak respiration), 1 μM carbonylcyanide-4-(trifluoromethoxy)-phenylhydrazone (FCCP) (maximal uncoupled respiration), 2.5 μM antimycin A and 1.25 μM rotenone (residual respiration). Experiments were performed with 8–10 wells per condition, and subsequently averaged. Oxygen consumption rate (OCR) values were adjusted for residual respiration and cell count using the CyQUANT Cell Proliferation Assay Kit (Thermo Fischer Scientific, Bleiswijk, Netherlands) according to the supplier’s protocol. To compare between plates, control routine respiration was set to 100.

### 4.4. Complex I and II-Stimulated Respiration

To assess mitochondrial respiration using NADH-producing and FADH_2_-producing substrates, mitochondrial respiration was measured using Seahorse respirometry in digitonin-permeabilized H9C2 cardiomyoblasts. Immediately prior to measurements, culture medium was replaced with MAS buffer (pH 7.4, 220 mM mannitol, 70 mM sucrose, 10 mM KH_2_PO_4_, 5 mM MgCl_2_, 2 mM HEPES, 1 mM EGTA, 4.0% fatty-acid free BSA). OCR was analyzed following a single injection of either 5 mM pyruvate and 2.5 mM malate (complex I-linked respiration), 10 mM succinate and 1.25 μM rotenone (complex II-linked respiration), or pyruvate, malate, succinate (complex I and II-linked respiration), together with 1 mM ADP and 25 μg/mL digitonin dissolved in MAS buffer at pH 7.4. Subsequently, 1.5 μM oligomycin was injected to assess leak respiration, followed by 2.5 μM antimycin A together with 1.25 μM rotenone for background respiration, which was subtracted from all values. Experiments were performed with 8–10 wells per condition, and subsequently averaged. OCR values were adjusted for cell count using the CyQUANT Cell Proliferation Assay Kit (Thermo Fischer Scientific, Bleiswijk, Netherlands) according to the supplier’s protocol.

### 4.5. Live-Cell Imaging

To assess mitochondrial morphology by live-cell imaging, H9C2 cardiomyoblasts were plated on coverslips as described above, rinsed with PBS and incubated in phenol-red-free DMEM with 200 nM tetramethylrhodamine methyl ester (TMRM, T5428 Sigma), which accumulates in polarized mitochondria, for a minimum of 20 min. Doxycycline was removed from the medium due to its interfering effects on fluorescence signals [52]. After wash-out, coverslips were placed inverted on slides. All images were obtained with a Leica TCS SP8 SMD, mounted on a Leica DMI6000 inverted microscope enclosed in an incubator at 37 °C. Images were processed using Leica LAS-X software, and ImageJ, using the FeatureJ plugin (ImageJ 1.45s; National Institutes of Health, Bethesda, USA). Particles were analyzed for area, aspect ratio and circularity as described before [10].

### 4.6. Substrate Flux Analysis

To study whether doxycycline alters metabolic flexibility and mitochondrial metabolism [34,49], we performed ^13^C-fluxomics as previously [53]. In short, the incubation was performed for 2 h in 500.000 cells/well, in DMEM without glucose, pyruvate, glutamine and phenol red (Life technologies, Bleiswijk, The Netherlands) supplemented with 50 μM oleic acid in 0.2% BSA, 1 mM glutamine, 50 μM carnitine, and 5 mM [^13^C_6_]-glucose (Cambridge Isotope Laboratories, Tewksbury, MA, USA). Every condition was performed in duplicate.

Cells were washed in saline twice, collected in ice-cold methanol-water, chloroform (1:1:2 *v*/*v*) and centrifuged at 10,000× *g* for 10 min. The aqueous phase of the extractions was collected and evaporated. The metabolite pellet was dissolved in 100 µL methanol-water (3:2 *v*/*v*) and analyzed by ultra-high-pressure liquid chromatography (Thermo Scientific, Waltman, MA, USA) with a SeQuant ZIC-cHILIC column (PEEK 100 × 2.1 mm, 3.0 µm particle size, Merck, Darmstadt, Germany) at 15 °C coupled to a Thermo Q Exactive (Plus) Orbitrap mass spectrometer (Thermo Scientific, Waltman, MA, USA). Data were acquired in negative-scan mode. Data analysis was performed in Xcalibur software (Thermo Scientific, Waltman, MA, USA). [^13^C]-label enrichment was calculated and corrected based on mass distribution isotopomer analysis [54].

### 4.7. Myocyte Isolation and Cytosolic Ca^2+^ Measurements

All animal handling and experiments were approved by the ethics committee of the Amsterdam UMC, and all procedures were in accordance with institutional and national and EU legislation.

Cytosolic Ca^2+^ signals were recorded as previously described [27]. Adult Wistar rats (~300 g; Charles River) were anaesthetized by isoflurane inhalation and killed by heart removal. Ventricular cardiomyocytes were isolated by enzymatic dissociation, and plated on laminin-coated dishes (MatTek Corporation, Ashland, MA, USA) for 1 h in M199 medium (PAA laboratories, Pasching, Austria) supplemented with 100 μg/mL ampicillin, 100 μg/mL carbenicillin and 5% fetal bovine serum (FBS). Cells were kept in culture for 2 days in M199 medium supplemented with 100 μg/mL ampicillin, 100 μg/mL carbenicillin, and insulin transferrin selenium (ITS; 0.2%), in increasing concentration doxycycline: 0, 10, or 30 μg/mL. On day 2, cardiomyocytes were loaded with 1 µM Fura-2 acetoxymethyl ester (Fura-2 AM, Life Technologies, Bleiswijk, The Netherlands) in Tyrode’s solution, containing NaCl (133.5 mM), KCl (5 mM), MgSO_4_ (1.2 mM), HEPES (10 mM), glucose (11.1 mM) and CaCl_2_ (1.8 mM; pH 7.4), for 15 min at room temperature, washed, and left for de-esterification for 15 min. Doxycycline was removed from the medium due to its interfering effects on fluorescence signals [52], and is known to be a calcium (and iron) chelator. A dual-beam excitation fluorescence photometry setup (IonOptix Corp. Milton, MA, USA), equipped with a HyperSwitch (IonOptix; LLC, Milton, MA, USA) was used for rapid wavelength switching between 340 and 380 nm. Excitation was measured at 510 nm at 250 Hz. The F340/F380 ratio was used as a measure of cytosolic Ca^2+^ concentration. Experiments were performed in a temperature-controlled dish at 37 °C equipped with platinum stimulation electrodes, filled with Tyrode’s solution. Bipolar pulses at 1 Hz were used for electrical field stimulation. Data were averaged per well to account for a different number of experiments performed per well and condition.To assess the arrhythmogenic potential of doxycycline, we first stressed other cardiomyocytes with a 2 Hz stimulation protocol for 20 s followed by 20 s at 4 Hz, and a resting period of 30 s. Cells were excluded from further analysis when they were arrhythmic before the start. Irregularities in contractions during the bout of 2 and 4 Hz, as well as the resting period, were confirmed by increased cytosolic calcium concentrations (such as calcium waves) and were counted during this fixed time.

### 4.8. Drosophila Melanogaster Stocks and Heart Wall Measurements

To assess doxycycline-induced arrhythmia and contractile dysfunction in *Drosophila melanogaster,* we used the *w^1118^* strain (Bloomington Drosophila Stock Center, IN, USA). All flies were maintained at 25 °C on standard medium [55]. Briefly, doxycycline (100 or 300 μg/mL) was dissolved in PBS and 0.2 mL was added to the medium during fertilization for 3 days [12]. Doxycycline concentrations were chosen based on a more rapid metabolism in Drosophila, and decay of doxycycline in PBS. Controls were subjected to PBS only. Prepupae were selected and placed on 1% agarose in PBS. Doxycycline was removed from the measurement medium. Heart wall contractions were measured utilizing high-speed digital video imaging (100 frames/second in triplicate periods of 20 s) by using a BlueFOX3 digital camera (Cytocypher, Amsterdam, Netherlands) on a Leica DM IL LED microscope with a 10× lens, followed by the generation of heart wall tracers. Tracers were used to determine cardiac parameters including heart rate and arrhythmicity index (defined as the standard deviation of the heart period) [56,57]. Time-lapse movies were collected and analyzed using the Kymograph plug-in in ImageJ to determine the heart rate, heart wall shortening (amplitude of diastolic and systolic heart wall contraction) and the duration of systole and diastole. Arrhythmicity index was calculated from 2 min.100 frames^−1^·s^−1^ high-speed movies with a 20× lens of the spontaneous heart wall contractions in prepupae and defined as the standard deviation of the heart period divided by the median heart period [57], by custom-made software. A higher arrhythmicity index is indicative of a higher heart rate variability.

### 4.9. Mice

All experimental procedures were in line with the guidelines from Directive 2010/63/EU of the European Parliament on the protection of animals used for scientific purposes, and national and institutional guidelines for animal welfare and approved by the ethical committee for animal experiments of the Amsterdam UMC. To assess whether the cardiac mitochondrial and contractile function in mice with diabetes were more susceptible to doxycycline toxicity, we used 10–12 weeks old male heterozygous C57BL/KsOlaHsd-Lepr^db^ (db/+; control) and homozygous C57BL/KsOlaHsd-Lepr^db^ (db/db, diabetes) mice from Envigo. All mice were housed at room temperature according to a 12 h light-dark cycle. Food and water were given ad libitum. Doxycycline (1.7 g·L^−1^) was supplemented to the drinking water, aiming at a daily concentration of 500 mg.kg^−1^.day^−1^, as previously used [12]. Amoxicillin (0.2 g/L; daily dosage: 50 mg.kg^−1^.day^−1^) was used as a control antibiotic to account for alterations in the microbiome. Total treatment was 2–3 weeks. Animals were sacrificed by cervical dislocation under 4% isoflurane inhalation anesthesia, and the heart and blood (serum) were rapidly sampled.

### 4.10. In Vivo Systolic and Diastolic Function

Cardiac function in 2–4% isoflurane-anaesthetized mice was assessed by cardiac MRI, using a 7.0 Tesla MR Solutions small animal scanner (MR Solutions, Guildford, UK) equipped with a 38-mm-diameter mouse volume coil. For left ventricle (LV) systolic function measurement, we used a cardio-respiratory gated multi-slice short-axis cine MRI acquisition using the following parameters: TR/TE = 7/2.8 ms, flip angle = 20°, FOV = 30 × 30 mm^2^, matrix size = 192 × 192, slice thickness = 1 mm, number of cardiac frames = 12–15, number of slices = 7, number of averages = 5, acquisition time = 20 min. To assess diastolic function, a single mid-ventricular short-axis slice was acquired using a high frame rate retrospectively gated cardiac sequence [58] with the following sequence parameters: TR/TE = 7/2.35 ms, flip angle = 15°, FOV: = 30 × 30mm^2^, matrix size = 192 × 192, slice thickness = 1 mm, number of k-space repetitions = 400, acquisition time = 13 min. Off-line reconstruction of the diastolic function measurements was performed in MATLAB R2019b (The Mathworks, Natick, MA, USA) using custom-built routines. In short, imaging data were binned into 60 frames and reconstructed by compressed sensing algorithms using Berkeley Advanced Reconstruction Toolbox (BART) [59,60]. All images were analyzed using MEDIS software (Medis, Leiden, The Netherlands). End-diastolic and end-systolic volumes of the left ventricle were used to calculate ejection fraction (EF) as a measure for systolic function. Diastolic function was measured as the ratio of the early or elastic (E’) and atrial (A’) filling rates.

### 4.11. Mitochondrial Respiration in Doxycycline-Treated Mice

As long-term isoflurane anesthetics are known to affect cardiac mitochondrial respiration [61], we sacrificed the mice on different days as the MRI imaging. Cardiac mitochondrial oxygen consumption was measured in the permeabilized tissue taken from the apex [26]. Thin bundles of cardiomyocytes were permeabilized with 50 µg·mL^−1^ saponin for 30 min at 4 °C in a solution containing (in mM) MES (50), taurine (20), imidazole (20), phosphocreatine (15), EGTA (7.2), MgCl_2_ (6.6), ATP (5.8), CaEGTA (2.8) and DTT (0.5), with pH set to 7.1. Tissue was subsequently washed in respiration solution, containing sucrose (110), K-lactobionate (60), taurine (20), HEPES (20), KH_2_PO_4_ (10), MgCl_2_ (3), EGTA (0.5), and 1 g·L^−1^ fatty acid-free BSA (pH 7.1), blotted dry, weighed and transferred to a respirometer (Oxygraph-2k; Oroboros Instruments, Innsbruck, Austria) in respiration solution at 37 °C. Oxygen concentration was maintained above 300 μM throughout the experiment to avoid limitations in oxygen supply.

Leak respiration was assessed after the addition of sodium glutamate (10 mM), sodium malate (0.5 mM) and sodium pyruvate (5 mM). NADH-linked (via complex I) respiration was measured after the addition of 2.5 mM ADP. Outer-mitochondrial membrane damage was tested by the addition of 10 μM cytochrome c and any increase in respiration of >20% was excluded from further analysis. Maximal NADH-linked respiration was assessed after the addition of cytochrome c, i.e., after alleviating possible effects of outer-membrane damage. Maximal OXPHOS capacity, with simultaneous input of electrons through complex I and II, was measured after the addition of 10 mM succinate. Maximal uncoupled respiration was measured after stepwise addition of 0.01 μM carbonylcyanide-4-(trifluoromethoxy)-phenylhydrazone (FCCP). Subsequently, succinate-linked respiration was measured after blocking complex I by the addition of 0.5 μM rotenone. Residual oxygen consumption was measured after the addition of antimycin A (2.5 μM) and was subtracted from all values. Respiration values were normalized to wet weight and expressed in pmol O_2_·s^−1^·mg^−1^. NADH-linked (Complex I) and succinate-linked (complex II) respiration were normalized to maximal respiration, to assess qualitative differences after accounting for differences in maximal oxidative capacity between groups.

### 4.12. Blood Parameters

Blood serum concentrations of glucose, non-esterified fatty acids and triglycerides were determined as described before [62].

### 4.13. Statistical Analysis

All data are presented as mean ± SEM, unless otherwise stated. Comparisons were made using one-way ANOVA, followed by Tukey’s post-hoc tests. Because the individual data from the particle analysis of the live-cell imaging showed large deviations from normal distribution, the median (area and aspect ratio) or 25% percentile values (circularity) were used for further between-group statistical analysis. A non-parametric Kruskal-Wallis (with Dunn’s comparisons) test was used for the median area. The level of significance was set at *P* < 0.05. Statistical analyses were performed using Prism 9 (GraphPad Software, Inc., La Jolla, CA, USA).

## Data Availability

Data is available upon request from corresponding authors.

## References

[1] Chen L., Knowlton A.A. (2010). Mitochondria and heart failure: New insights into an energetic problem. Minerva Cardioangiol..

[2] Nickel A., Löffler J., Maack C. (2013). Myocardial energetics in heart failure. Basic Res. Cardiol..

[3] Wüst R.C.I., Helmes M., Stienen G.J.M. (2015). Rapid changes in NADH and flavin autofluorescence in rat cardiac trabeculae reveal large mitochondrial complex II reserve capacity. J. Physiol..

[4] Murphy E., Ardehali H., Balaban R.S., DiLisa F., Dorn G.W., Kitsis R.N., Otsu K., Ping P., Rizzuto R., Sack M.N. (2016). Mitochondrial Function, Biology, and Role in Disease: A Scientific Statement from the American Heart Association. Circ. Res..

[5] Glancy B., Hartnell L.M., Combs C.A., Fenmou A., Sun J., Murphy E., Subramaniam S., Balaban R.S. (2017). Power Grid Protection of the Muscle Mitochondrial Reticulum. Cell Rep..

[6] Corliss J.O., Margulis L. (1972). Origin of Eukaryotic Cells. Trans. Am. Microsc. Soc..

[7] Scarpulla R.C. (2008). Transcriptional Paradigms in Mammalian Mitochondrial Biogenesis and Function. Physiol. Rev..

[8] Schon E.A., DiMauro S., Hirano M. (2012). Human mitochondrial DNA: Roles of inherited and somatic mutations. Nat. Rev. Genet..

[9] Couvillion M.T., Soto I.C., Shipkovenska G., Churchman L.S. (2016). Synchronized mitochondrial and cytosolic translation programs. Nat. Cell Biol..

[10] Houtkooper R.H., Mouchiroud L., Ryu D., Moullan N., Katsyuba E., Knott G., Williams R.W., Auwerx J. (2013). Mitonuclear protein imbalance as a conserved longevity mechanism. Nat. Cell Biol..

[11] Clark-Walker G.D., Linnane A.W. (1966). In vivo differentiation of yeast cytoplasmic and mitochondrial protein synthesis with antibiotics. Biochem. Biophys. Res. Commun..

[12] Moullan N., Mouchiroud L., Wang X., Ryu D., Williams E.G., Mottis A., Jovaisaite V., Frochaux M.V., Quiros P.M., Deplancke B. (2015). Tetracyclines Disturb Mitochondrial Function across Eukaryotic Models: A Call for Caution in Biomedical Research. Cell Rep..

[13] Tischner C., Hofer A., Wulff V., Stepek J., Dumitru I., Becker L., Haack T., Kremer L., Datta A.N., Sperl W. (2014). MTO1 mediates tissue specificity of OXPHOS defects via tRNA modification and translation optimization, which can be bypassed by dietary intervention. Hum. Mol. Genet..

[14] Wang J., Wilhelmsson H., Graff C., Li H., Oldfors A., Rustin P., Brüning J.C., Kahn C.R., Clayton D.A., Barsh G.S. (1999). Dilated cardiomyopathy and atrioventricular conduction blocks induced by heart-specific inactivation of mitochondrial DNA gene expression. Nat. Genet..

[15] Galmiche L., Serre V., Beinat M., Assouline Z., Lebre A.-S., Chretien D., Nietschke P., Benes V., Boddaert N., Sidi D. (2011). Exome sequencing identifies MRPL3 mutation in mitochondrial cardiomyopathy. Hum. Mutat..

[16] Bray A.W., Ballinger S.W. (2017). Mitochondrial DNA mutations and cardiovascular disease. Curr. Opin. Cardiol..

[17] Duewelhenke N., Krut O., Eysel P. (2006). Influence on Mitochondria and Cytotoxicity of Different Antibiotics Administered in High Concentrations on Primary Human Osteoblasts and Cell Lines. Antimicrob. Agents Chemother..

[18] Muanda F.T., Sheehy O., Berard A. (2017). Use of antibiotics during pregnancy and the risk of major congenital malformations: A population based cohort study. Br. J. Clin. Pharmacol..

[19] Mosholder A.D., Lee J.-Y., Zhou E.H., Kang E.M., Ghosh M., Izem R., Major J.M., Graham D.J. (2017). Long-Term Risk of Acute Myocardial Infarction, Stroke, and Death With Outpatient Use of Clarithromycin: A Retrospective Cohort Study. Am. J. Epidemiol..

[20] Ray W.A., Murray K.T., Hall K., Arbogast P.G., Stein C.M. (2012). Azithromycin and the Risk of Cardiovascular Death. N. Engl. J. Med..

[21] Meijer C.A., Stijnen T., Wasser M.N., Hamming J.F., van Bockel J.H., Lindeman J.H. (2013). Pharmaceutical Aneurysm Stabilisation Trial Study, G. Doxycycline for stabilization of abdominal aortic aneurysms: A randomized trial. Ann. Intern. Med..

[22] Wüst R.C., Houtkooper R.H., Auwerx J. (2020). Confounding factors from inducible systems for spatiotemporal gene expression regulation. J. Cell Biol..

[23] Adadevoh B.K., Ogunnaike I.A., Bolodeoku J.O. (1976). Serum levels of doxycycline in normal subjects after a single oral dose. BMJ.

[24] Agwuh K.N., MacGowan A. (2006). Pharmacokinetics and pharmacodynamics of the tetracyclines including glycylcyclines. J. Antimicrob. Chemother..

[25] Blanchard P., Rudhardt M., Fabre J. (1975). Behaviour of Doxycycline in the Tissues. Chemotherapy.

[26] Wüst R.C., De Vries H.J., Wintjes L.T., Rodenburg R.J., Niessen H.W., Stienen G.J. (2016). Mitochondrial complex I dysfunction and altered NAD(P)H kinetics in rat myocardium in cardiac right ventricular hypertrophy and failure. Cardiovasc. Res..

[27] Wüst R.C.I., Helmes M., Martin J.L., Van Der Wardt T.J.T., Musters R.J.P., Van Der Velden J., Stienen G.J.M. (2017). Rapid frequency-dependent changes in free mitochondrial calcium concentration in rat cardiac myocytes. J. Physiol..

[28] Dieteren C.E., Willems P.H., Swarts H.G., Fransen J., Smeitink J.A., Koopman W.J., Nijtmans L.G. (2011). Defective mitochondrial translation differently affects the live cell dynamics of complex I subunits. Biochim. Biophys. Acta (BBA) Bioenerg..

[29] Lim S.C., Hroudová J., Van Bergen N.J., Sanchez M.I.G.L., Trounce I.A., McKenzie M. (2016). Loss of mitochondrial DNA-encoded protein ND1 results in disruption of complex I biogenesis during early stages of assembly. FASEB J..

[30] Ingwall J.S. (2008). Energy metabolism in heart failure and remodelling. Cardiovasc. Res..

[31] Nickel A., Kohlhaas M., Maack C. (2014). Mitochondrial reactive oxygen species production and elimination. J. Mol. Cell. Cardiol..

[32] Anan R., Nakagawa M., Miyata M., Higuchi I., Nakao S., Suehara M., Osame M., Tanaka H. (1995). Cardiac involvement in mitochondrial diseases. A study on 17 patients with documented mitochondrial DNA defects. Circulation.

[33] Houten S.M., Violante S., Ventura F.V., Wanders R.J. (2016). The Biochemistry and Physiology of Mitochondrial Fatty Acid beta-Oxidation and Its Genetic Disorders. Annu. Rev. Physiol..

[34] Smith R.L., Soeters M.R., Wüst R.C.I., Houtkooper R.H. (2018). Metabolic Flexibility as an Adaptation to Energy Resources and Requirements in Health and Disease. Endocr. Rev..

[35] Sequeira V., Najafi A., McConnell M., Fowler E.D., Bollen I.A.E., Wüst R.C.I., Dos Remedios C., Helmes M., White E., Stienen G.J.M. (2015). Synergistic role of ADP and Ca2+in diastolic myocardial stiffness. J. Physiol..

[36] Inesi G., Lewis D., Ma H., Prasad A., Toyoshima C. (2006). Concerted Conformational Effects of Ca2+and ATP Are Required for Activation of Sequential Reactions in the Ca2+ATPase (SERCA) Catalytic Cycle†,‡. Biochemistry.

[37] Macdonald W.A., Stephenson D.G. (2001). Effects of ADP on sarcoplasmic reticulum function in mechanically skinned skeletal muscle fibres of the rat. J. Physiol..

[38] Fowler E.D., Benoist D., Drinkhill M.J., Stones R., Helmes M., Wüst R.C., Stienen G.J., Steele D.S., White E.T. (2015). Decreased creatine kinase is linked to diastolic dysfunction in rats with right heart failure induced by pulmonary artery hypertension. J. Mol. Cell. Cardiol..

[39] Taguchi T., Ishihara N., Jofuku A., Oka T., Mihara K., Nakagawa T., Uozumi N., Nakano M., Mizuno-Horikawa Y., Okuyama N. (2007). Mitotic Phosphorylation of Dynamin-related GTPase Drp1 Participates in Mitochondrial Fission. J. Biol. Chem..

[40] Yang K.-C., Bonini M.G., Dudley S.C. (2014). Mitochondria and arrhythmias. Free. Radic. Biol. Med..

[41] Zhang Y., Guallar E., Ashar F.N., Longchamps R.J., A Castellani C., Lane J., Grove M.L., Coresh J., Sotoodehnia N., Ilkhanoff L. (2017). Association between mitochondrial DNA copy number and sudden cardiac death: Findings from the Atherosclerosis Risk in Communities study (ARIC). Eur. Heart J..

[42] Wiersma M., Van Marion D.M., Wüst R.C., Houtkooper R.H., Zhang D., De Groot N.M., Henning R.H., Brundel B.J. (2019). Mitochondrial Dysfunction Underlies Cardiomyocyte Remodeling in Experimental and Clinical Atrial Fibrillation. Cells.

[43] Brown D.A., O’Rourke B. (2010). Cardiac mitochondria and arrhythmias. Cardiovasc. Res..

[44] Zima A.V., Blatter L.A. (2006). Redox regulation of cardiac calcium channels and transporters. Cardiovasc. Res..

[45] Cerisano G., Buonamici P., Valenti R., Sciagra R., Raspanti S., Santini A., Carrabba N., Dovellini E.V., Romito R., Pupi A. (2014). Early short-term doxycycline therapy in patients with acute myocardial infarction and left ventricular dysfunction to prevent the ominous progression to adverse remodelling: The TIPTOP trial. Eur. Heart J..

[46] Castro M.M., Tanus-Santos J.E., Gerlach R.F. (2011). Matrix metalloproteinases: Targets for doxycycline to prevent the vascular alterations of hypertension. Pharmacol. Res..

[47] Ortiz-Vilchis P., Yamazaki K.G., Rubio-Gayosso I., Ramírez-Sánchez I., Calzada C., Romero-Perez D., Ortiz A., Meaney E., Taub P., Villarreal F. (2014). Co-administration of the flavanol (-)-epicatechin with doxycycline synergistically reduces infarct size in a model of ischemia reperfusion injury by inhibition of mitochondrial swelling. Eur. J. Pharmacol..

[48] Chen Y., Chen Y., Wang N., Gu S., Wang M., Fu Y., Wei C., Xu W. (2021). Doxycycline in Extremely Low Dose Improves Glycemic Control and Islet Morphology in Mice Fed a High-Fat Diet. Diabetes Metab. Syndr. Obes. Targets Ther..

[49] Makrecka-Kuka M., Liepinsh E., Murray A.J., Lemieux H., Dambrova M., Tepp K., Puurand M., Käämbre T., Han W.H., De Goede P. (2019). Altered mitochondrial metabolism in the insulin-resistant heart. Acta Physiol..

[50] Miranda-Silva D., Wüst R.C.I., Conceição G., Gonçalves-Rodrigues P., Gonçalves N., Gonçalves A., Kuster D.W.D., Leite-Moreira A.F., Van Der Velden J., Beleza J.M.D.S. (2019). Disturbed cardiac mitochondrial and cytosolic calcium handling in a metabolic risk-related rat model of heart failure with preserved ejection fraction. Acta Physiol..

[51] Knottnerus S.J.G., Mengarelli I., Wüst R.C.I., Baartscheer A., Bleeker J.C., Coronel R., Ferdinandusse S., Guan K., Li W., Luo X. (2020). Electrophysiological Abnormalities in VLCAD Deficient hiPSC-Cardiomyocytes Can Be Improved by Lowering Accumulation of Fatty Acid Oxidation Intermediates. Int. J. Mol. Sci..

[52] Khader H., Solodushko V., Al-Mehdi A.B., Audia J., Fouty B. (2013). Overlap of Doxycycline Fluorescence with that of the Redox-Sensitive Intracellular Reporter roGFP. J. Fluoresc..

[53] Alves T.C., Pongratz R.L., Zhao X., Yarborough O., Sereda S.B., Shirihai O.S., Cline G.W., Mason G.F., Kibbey R.G. (2015). Integrated, Step-Wise, Mass-Isotopomeric Flux Analysis of the TCA Cycle. Cell Metab..

[54] Fernández-Fernández M., Rodríguez-González P., Garcia-Alonso J.I. (2016). A simplified calculation procedure for mass isotopomer distribution analysis (MIDA) based on multiple linear regression. J. Mass Spectrom..

[55] Wiersma M., Meijering R.A.M., Qi X., Zhang D., Liu T., Hoogstra-Berends F., Sibon O.C.M., Henning R.H., Nattel S., Brundel B.J.J.M. (2017). Endoplasmic Reticulum Stress Is Associated With Autophagy and Cardiomyocyte Remodeling in Experimental and Human Atrial Fibrillation. J. Am. Heart Assoc..

[56] Viswanathan M.C., Kaushik G., Engler A.J., Lehman W., Cammarato A. (2014). A Drosophila melanogaster model of diastolic dysfunction and cardiomyopathy based on impaired troponin-T function. Circ. Res..

[57] Fink M., Callol-Massot C., Chu A., Ruiz-Lozano P., Belmonte J.C.I., Giles W., Bodmer R., Ocorr K. (2009). A new method for detection and quantification of heartbeat parameters in Drosophila, zebrafish, and embryonic mouse hearts. Biotechniques.

[58] Coolen B.F., Abdurrachim D., Motaal A.G., Nicolay K., Prompers J.J., Strijkers G.J. (2012). High frame rate retrospectively triggered Cine MRI for assessment of murine diastolic function. Magn. Reson. Med..

[59] Motaal A.G., Coolen B.F., Abdurrachim D., Castro R.M., Prompers J.J., Florack L.M.J., Nicolay K., Strijkers G.J. (2012). Accelerated high-frame-rate mouse heart cine-MRI using compressed sensing reconstruction. NMR Biomed..

[60] Uecker M., Ong F., Tamir J., Bahri D., Virtue P., Cheng J., Zhang T., Lustig M. (2015). Berkeley Advanced Reconstruction Toolbox. Proc. Intl. Soc. Mag. Reson. Med..

[61] Kishikawa J.-I., Inoue Y., Fujikawa M., Nishimura K., Nakanishi A., Tanabe T., Imamura H., Yokoyama K. (2018). General anesthetics cause mitochondrial dysfunction and reduction of intracellular ATP levels. PLoS ONE.

[62] Bakermans A.J., Boekholdt S.M., de Vries D.K., Reckman Y.J., Farag E.S., de Heer P., Uthman L., Denis S.W., Zuurbier C.J., Houtkooper R.H. (2021). Quantification of Myocardial Creatine and Triglyceride Content in the Human Heart: Precision and Accuracy of in vivo Proton Magnetic Resonance Spectroscopy. J. Magn. Reson. Imaging.

